# Supplementary material for: Rapid turnover of life-cycle-related genes in the brown algae
Source: Genome Biol. 2019 Feb 14;20:35. doi: 10.1186/s13059-019-1630-6 (PMC6374913; doi:10.1186/s13059-019-1630-6)
Supplement: Supplementary file 3 — Protein alignments, corresponding Gblocks html file and CDS sequences for the lineage conserved gametophyte-biased genes. (ZIP 2109 kb) [file 13059_2019_1630_MOESM3_ESM.zip › Con_GA_alignments/Ec-12_000390.aa_ali.fasta-gb.htm]

Ec-12\_000390.aa.fasta.trans.aa\_ali.fasta


## Gblocks 0.91b Results

Processed file: **Ec-12\_000390.aa.fasta.trans.aa\_ali.fasta**  
Number of sequences: **2**  
Alignment assumed to be: **Protein**  
New number of positions: **749** (selected positions are underlined in blue)

```
                         10        20        30        40        50        60
                 =========+=========+=========+=========+=========+=========+
Ec-12_000390     MSGRARVPLGASERAGIFGNPSSARTAPSTLSDLQSTHRDDARRDSVPRAISRQHQHQHQ
MSTRG.33749.1    MSGRARVPLGATERSGIFGNPTSARTAPSSEADLRS-QTEDSRRGDVPRADSRQPQQQQA
                 ####################################    ####################


                         70        80        90       100       110       120
                 =========+=========+=========+=========+=========+=========+
Ec-12_000390     GEAVAGSASATPYGVLSPGRRRPYCSPYNGNPITPGREDFGPTSQSSREGGAAAGKEGPG
MSTRG.33749.1    G---AGSAKATPYGALSPGRRRPFSSPYNGNPIAPGRAENGPTNQGSQEGSRPDRQDGSQ
                 #   ######################################################  


                        130       140       150       160       170       180
                 =========+=========+=========+=========+=========+=========+
Ec-12_000390     -EPVRSEEPREQNAFGGNIGEPDGGGGDLLELEHIIGYTGHFAQTIIAHPEDKGVMIKSV
MSTRG.33749.1    QESSRNEDLGAKSIVETTNGARDDED-NMLELEHIIGYTGHYSQTVLAHPGGSNVMIKSV
                                              ###############################


                        190       200       210       220       230       240
                 =========+=========+=========+=========+=========+=========+
Ec-12_000390     GALLVTGSISDPHQQHLLRGHDMEISALTVSSSGALIATGQRGSTKVKGYPAPVFLWDYK
MSTRG.33749.1    GALLVIGSMTDSHRQSLLRGHDMEISALALSPSGSLIATGQLGSTRVKGYPAPVFLWDYA
                 ############################################################


                        250       260       270       280       290       300
                 =========+=========+=========+=========+=========+=========+
Ec-12_000390     TRTSLFVYQGLTECVCHLSFSPDARFLLGCGGDGLVYVWDTSTGELVTGKQYAEPSTLGE
MSTRG.33749.1    TRTSLFVFQGLTQGVRHLSFSPDQKFLLGCGGDRLVYVWDTSTGELVTGKQYSEPSTLGE
                 ############################################################


                        310       320       330       340       350       360
                 =========+=========+=========+=========+=========+=========+
Ec-12_000390     WTAVNLAGRRPVYEICLCTGNDVLALELRFDPSRRQWAISGGPMATPSSGLRRQYKCSAL
MSTRG.33749.1    WTSVKLSGRTPIYEICLCTGADLFALELSFDPSRRQWTITGGPMATPSSGLRRSYNCSAL
                 ############################################################


                        370       380       390       400       410       420
                 =========+=========+=========+=========+=========+=========+
Ec-12_000390     LPGGGFLLCGTDVGDMVVFGVNAKVFRASVPVCSGGMLSVCTDHSTGAVFCGGGNGIIRK
MSTRG.33749.1    LPGGNFLLCGTDVGDMVVFGVDARVFRASVPICSAGMLSICTDHATGAVYCGGGNGVIRK
                 ############################################################


                        430       440       450       460       470       480
                 =========+=========+=========+=========+=========+=========+
Ec-12_000390     VVGADMVWQQELETRLDGGVVSLSIVAGSGELLAGTQEGSVYRLLLADLSASKVSSSQRS
MSTRG.33749.1    IVGADMVWHQEMETTLDGGVVSLSILVDSCELLAGTQEGSVYRLLLADLSASKVSSSQRS
                 ############################################################


                        490       500       510       520       530       540
                 =========+=========+=========+=========+=========+=========+
Ec-12_000390     PVLSVACGDRCDAFATCAQDGAVRIWELCDFGVVAEASERSQRSAGSARALFLCWLGDEA
MSTRG.33749.1    PILSVACGSRCDAFATCAQDGTVRVWDLCDFGVVAEASERPQGAAYGARSQFLCWLGEEA
                 ############################################################


                        550       560       570       580       590       600
                 =========+=========+=========+=========+=========+=========+
Ec-12_000390     VVTGWSDCSVRCYDAATGALRWAIPKAHRAAVTCCAVRFDSSASFLVTGAEDGSVSVWSL
MSTRG.33749.1    VVTGWSDCSVRCHDAATGVLRWAIPKAHRAPVTCCAVSFGPSASFLVTGAEDGSVSVWEL
                 ############################################################


                        610       620       630       640       650       660
                 =========+=========+=========+=========+=========+=========+
Ec-12_000390     RTRELLLRFSEHQKGVTTVLVDVASPNLVHSAGTDCTVLTYDLRKEKRTVAHMTRGGAFQ
MSTRG.33749.1    RTRELLLRFSEHQKGVRSVLVDVASPNLVHSAGSDCTVFTYDLRKERRTVAHMTRAGAFQ
                 ############################################################


                        670       680       690       700       710       720
                 =========+=========+=========+=========+=========+=========+
Ec-12_000390     GMTQRIDSENELITCDIHGRLLTWDCDYPAPVQAMQDPSKQRLSCVSVSPSGRYMALGGD
MSTRG.33749.1    SMTQRKDSENELITCDLHGRLLTWDCDYRDPVQVMQDPSKQRLSCVNVSPSGRYMALGGD
                 ############################################################


                        730       740       750       760       770       780
                 =========+=========+=========+=========+=========+=========+
Ec-12_000390     DELVKVLDLATNCETVACGRAHAGAITSVQWTRDERQLISAAEDCTLAIWNFFG-SPPPP
MSTRG.33749.1    DETIKVLDLANDSETVAYGQAHAGAITSVQWTRDERQLISAADDCTLAIWNFFGPSGSPP
                 ###################################################### #####


                        790
                 =========+
Ec-12_000390     SGCPDEQRKP
MSTRG.33749.1    SGRSDVRRA-
                 ########
```

```
Parameters used
Minimum Number Of Sequences For A Conserved Position: 2
Minimum Number Of Sequences For A Flanking Position: 2
Maximum Number Of Contiguous Nonconserved Positions: 8
Minimum Length Of A Block: 10
Allowed Gap Positions: None
Use Similarity Matrices: Yes
```

```
Flank positions of the 5 selected block(s)
Flanks: [1  36]  [41  61]  [65  118]  [150  774]  [776  788]  

New number of positions in Ec-12_000390.aa.fasta.trans.aa_ali.fasta-gb:  749  (94% of the original 790 positions)
```
